# Exploring the Impact of Bio-Based Plasticizers on the Curing Behavior and Material Properties of a Simplified Tire-Tread Compound

**DOI:** 10.3390/polym16131880

**Published:** 2024-07-01

**Authors:** Frances van Elburg, Fabian Grunert, Claudia Aurisicchio, Micol di Consiglio, Raffaele di Ronza, Auke Talma, Pilar Bernal-Ortega, Anke Blume

**Affiliations:** 1 Elastomer Technology & Engineering, Department of Mechanics of Solids, Surfaces & Systems (MS3), Faculty of Engineering Technology, University of Twente, Drienerlolaan 5, 7522 NB Enschede, The Netherlands; f.grunert@utwente.nl (F.G.); a.g.talma-1@utwente.nl (A.T.); m.d.p.bernalortega@utwente.nl (P.B.-O.); a.blume@utwente.nl (A.B.); 2 Italian Branch–Technical Center, Bridgestone EU NV/SA, Via del Fosso del Salceto 13/15, 00128 Rome, Italy; claudia.aurisicchio@bridgestone.eu (C.A.);

**Keywords:** TDAE, bio-based plasticizers, sustainability, tire tread, silica-filled compound, sunflower oil, coconut oil, cardanol

## Abstract

The tire industry needs to become more sustainable to reduce pollution and fight climate change. Replacing fossil ingredients in a tire-tread compound with bio-based alternatives is an approach to create a more sustainable product. For instance, the plasticizer can be replaced, which is a petroleum-based ingredient used in relatively high amounts in the rubber. This approach was followed in the current study. Three plant-based plasticizers were selected as potential substitutes for treated distillate aromatic extract (TDAE) in a simplified tire-tread compound formulation, namely, sunflower oil, coconut oil, and cardanol. Additionally, squalane was used as a TDAE replacement to further investigate the possible interactions between plasticizers and other compound ingredients. Squalane (C_30_H_62_) is a fully saturated substance, containing six methyl groups but no additional chemical functional groups. Therefore, it was expected that squalane would result in limited interactions within the studied system. All alternatives to TDAE showed an increased cure rate and decreased scorch time, except squalane. This indicates that the three bio-based plasticizers might interact with the vulcanization system. For example, they could function as an additional coactivator of the curing system and/or shield the silica surface. A severe decrease in maximum torque and an increase in elongation at break were obtained for cardanol and sunflower oil. Both plasticizers also resulted in lower crosslink densities compared to the other compounds. A model study with the bio-plasticizers and sulfur verified that the unsaturation in the cardanol and sunflower oil reacted with the crosslinking agent. This leads to less sulfur available for the curing reaction, explaining the low maximum torque. The tan δ curves showed that all replacements resulted in a decrease in the glass transition temperature of the compound. Although all oil alternatives displayed promising results, none of them are suitable as a direct substitute for TDAE in a tire-tread compound due to its ability to interact additionally with other rubber ingredients and contribute in this form to the reinforcement of the compound.

## 1. Introduction

In recent years, one of the main goals of the tire industry has been to become more sustainable to prevent and reduce climate change. Members of eleven leading tire companies created a roadmap [1] with specific guidelines to achieve this goal. An example of an opportunity stated in the roadmap is the sustainable usage of natural resources as an alternative to fossil-based ingredients [1]. One petroleum-based ingredient often used, and in relatively large quantities, in tire-tread compounds is treated distillate aromatic extract (TDAE). TDAE is an aromatic oil, consisting of 25 wt% aromatic carbon, 30 wt% naphthenic carbon and 45 wt% paraffinic carbon [2]. However, the exact chemical structure of TDAE is difficult to determine. This makes the finding of bio-based alternatives with similar chemical structures and functional groups to TDAE challenging. 

Oil is added to a rubber compound to function as an extender, softener and plasticizer. It, for instance, reduces the viscosity, which improves the processability of the compound. Furthermore, due to the addition of oil, higher filler loadings can be used. However, they do influence the final properties of the compound. For example, the addition of oils to rubber compounds results in lower glass transition temperatures and improved low-temperature flexibility [3]. This influence can be explained by the free volume theory [3,4]. Oil migrates into the free volume between polymer chains and expands this volume. Due to the increased volume, the polymer has more space to rotate and move, increasing its mobility. Therefore, the internal friction in the compound is reduced, influencing the material properties [3].

To obtain an indication of a chemical substance that could be a suitable replacement for TDAE in the used rubber compound, the Hansen solubility parameter (HSP) can be calculated. This parameter gives insight into the compatibility between two or more substances. The HSP is based on Hildebrand’s theory, which states that the solubility parameter is related to the energy of vaporization divided by the molar volume of a substance. To obtain the HSP value, the solubility parameters of three parts are calculated, namely, the dispersion, polar and hydrogen parts. If these three parts are similar for two separate substances, the substances are thermodynamically compatible with each other [5]. If the new oil replacement has an HSP value similar to TDAE, it is likely that they are compatible and that the new oil is compatible with the used polymers as well. However, compatibility is only one of the many characteristics that influence the performance of the plasticizer in the compound. Other important properties are, for instance, the volatility and viscosity of the oil [6]. 

Multiple plant-based plasticizers have been investigated in various rubber compounds [7]. For example, Flanigan et al. [8] investigated palm oil, cashew nutshell liquid, flaxseed oil and soybean oil in silica-filled S-SBR compounds. Alexander and Thachil [9] researched the influence of cardanol in carbon-black-filled NR. Othman and Ismail [10] investigated different concentrations of coconut oil in an NR compound filled with carbon black. Sunflower oil and soybean oil were studied by Ezzoddin et al. [11] in carbon-black-filled SBR. Focusing on tire-tread compounds, Flanigan et al. [12] researched tall oil, linseed oil, castor oil, orange oil and vulcanized vegetable oil in a silica-filled SBR/BR tread compound. In addition, Siriwong et al. [13] studied soybean oil in a tire-tread compound based on a carbon-black-filled SBR/BR blend. However, all these studies show that the bio-based alternatives result in trade-offs in the material properties. Further, there is still a lot unknown about the plasticizing mechanism of the bio-based replacements in the rubber compounds. For example, the influences of sunflower oil, coconut oil and cardanol in a silica-filled SBR/BR compound and the interactions between these bio-based oils and the other rubber ingredients have not yet been researched in detail. 

In this work, the influence of sunflower oil, coconut oil and cardanol on a simplified tire-tread compound is investigated, focusing on the cure behavior and material properties of the rubber compound. In addition to the plant-based plasticizers, squalane is used to replace TDAE. Squalane is a saturated substance of carbon and hydrogen atoms with six methyl groups and without additional functional chemical groups. Therefore, it is assumed that the interactions between squalane and other ingredients in the rubber compounds are limited. Comparing the results of squalane with the results obtained for the TDAE, it should be possible to prove if TDAE only acts as a plasticizer or if this petroleum-based oil does also interact with other rubber ingredients. All four plasticizer alternatives are compared to a reference compound containing TDAE.

## 2. Materials and Methods

### 2.1. Materials

A rubber blend of S-SBR (SPRINTAN^®^ SLR 4601, styrene content 21%, molecular weight = 450kg/mol) (Synthos Schkopau GMBH, Schkopau, Germany) and BR (Buna^®^ CB24, cis-1,4 content > 96%, molecular weight = 620 kg/mol) (Arlanxeo, Dormagen, Germany) was used. The compound was filled with highly reinforcing silica, ULTRASIL^®^ 7000 GR (specific surface area (CTAB) 160 m^2^/g, Evonik Industries, Wesseling, Germany). As a silane coupling agent, bis(triethoxysilylpropyl) disulfide (TESPD) (Evonik Industries, Wesseling, Germany) was used. Zinc oxide (ZnO) (Umicore zinc Chemicals, Angleur, Belgium) and stearic acid (Emery Oleochemicals GmbH, Düsseldorf, Germany) were added as activators. Furthermore, the curing package consisted of sulfur (Zolfindustria, Trecate, Italy), N-tert-butyl-benzothiazole sulfonamide (TBBS) (General Química S.A., Lantarón, Spain) and N,N′-diphenylguanidine (DPG) (MLPC International (Arkema Group), Rion-des-Landes, France).

TDAE (Hansen & Rosenthal, Hamburg, Germany) was used as oil in the reference compound. The bio-based plasticizers analyzed in this study were Cardanol NX 2024 (Equilex, Schiedam, The Netherlands), sunflower oil and coconut oil (local market, Enschede, The Netherlands). Finally, another substance used as a plasticizer in this study was squalane (Thermo Fisher Scientific, Geel, Belgium). Table 1 shows the molecular weight (Mw), the density (ρ), the Hansen solubility parameter (HSP), the chemical structure and the melting point (MP) of the plasticizers. The latter term “melting point” is used to describe the temperature at which a given solid oil changes from a solid to a liquid state.

### 2.2. Compounding and Mixing

#### 2.2.1. Rubber Formulation

Table 2 shows the used rubber formulation for the studied compounds. Due to similarities in densities, TDAE was replaced with the four different plasticizers in a 1:1 ratio.

#### 2.2.2. Mixing Procedure

The compounds were mixed with the Brabender Plasticorder 350S (Duisburg, Germany), according to the mixing procedure shown in Table 3. The chamber volume of this internal mixer is 390 cm^3^. After each mixing stage, the materials were sheeted out on a two-roll mill. 

### 2.3. Analytical Testing

#### 2.3.1. Vulcanization

To obtain the optimal vulcanization time, the Rubber Process Analyzer TA Elite (TA Instruments, New Castle, DE, USA) was used. A frequency of 1.667 Hz and deformation of 6.98% were applied, and the temperature was set to 160 °C. The curing behavior was measured for 1 h. Subsequently, the time to reach 90% of conversion (t90) was used to cure the 2 mm thick test samples for the stress–strain and dynamic mechanical analysis measurements. The rubber samples were vulcanized at a temperature of 160 °C and under a pressure of 100 bar in a Wickert WLP 1600 hydraulic press (Wickert, Landau in der Pfalz, Germany).

#### 2.3.2. Cured Payne Effect

The Rubber Process Analyzer TA Elite (TA Instruments, New Castle, DE, USA) was used to obtain the cured Payne effect. Samples were first cured at 160 °C in the device, according to their t90. Directly after this, the storage modulus (G′) was measured at 100 °C. Stain sweeps from 0.56% to 100% were applied to the cured samples, using a frequency of 1.667 Hz.

#### 2.3.3. Equilibrium Swelling

The crosslink densities of the compounds were measured by the equilibrium swelling method. Cured samples were first extracted in acetone using a Soxhlet extractor to remove the soluble ingredients, like the plasticizer. Afterward, the samples were swollen in toluene for 7 days. The crosslink density was calculated with the Flory–Rehner equation [15]:

$$\nu =-\frac{\ln\left(1-V_{r}\right)+V_{r}+\chi V_{r}^{2}}{V_{0}\left({V_{r}}^{\frac{1}{3}}-\frac{{2V}_{r}}{f}\right)}$$

where

V_r_ = the volume percentage/fraction of rubber in a swollen sample
V_0_ = the molar volume of the solvent used (106.9 cm^3^/mol for toluene)
f = the functionality of crosslinks (assuming that tetra-functional crosslinks are formed, f = 4)
χ = the Flory–Huggins rubber–solvent interaction parameter (for SBR in toluene, χ = 0.378 [16])
v = the crosslink density per unit volume (mol/cm^3^)

#### 2.3.4. Stress–Strain Behavior

Using the universal mechanical tester Zwick Z01 (Zwick, Ulm, Germany) according to ISO 37 [17] (die type 2), the stress–strain behavior of the compounds was measured. A crosshead speed of 500 mm/min was used. The tensile test was performed at room temperature. Five samples of each compound were measured, and the average values of these five measurements were analyzed. Additionally, the median of each compound was displayed in a stress–strain graph.

#### 2.3.5. Dynamical Properties 

Dynamic mechanical analysis was performed with the Gabo-Netzsch Eplexor tester (Netzsch, Germany). Rectangular-shaped samples with a thickness of 2 mm and a width of 5.5 mm were measured in tension mode. A static strain of 0.3% and a dynamic strain of 0.1% were used. The test was carried out with a dynamic load frequency of 10 Hz over a temperature range from −80 to 80 °C.

## 3. Results and Discussion

### 3.1. Rubber Compounds

#### 3.1.1. Vulcanization

**Figure 1:**
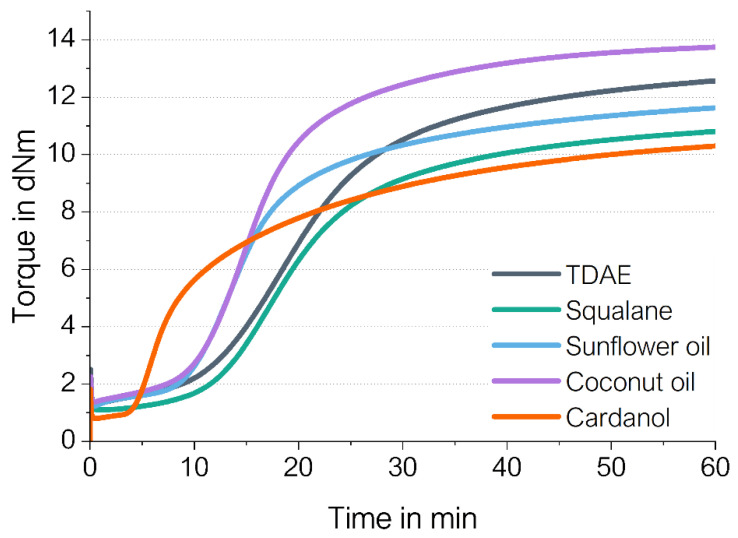
Cure curves of the studied compounds.

Figure 1 shows the cure curves of the plasticizer alternatives compared to the reference with TDAE oil. If all substances act only as plasticizers, their cure curve progression should be similar. However, different curing behaviors were obtained for the different plasticizers, indicating that the substances do influence the curing process. Squalane results in the most similar curing behavior as TDAE, with the same optimum cure time (t90) and scorch time (t2), as shown in Table 4. The table also indicates that squalane results in a lower minimum torque (ML) and a lower maximum torque (MH) compared to TDAE, so differences can be distinguished as well. Furthermore, the table shows that the crosslink density (CLD) evaluated with equilibrium swelling is slightly lower for squalane in comparison with TDAE. These differences in the cure curves of squalane and TDAE indicate that TDAE does have an influence on the vulcanization of the compound and that it interacts with other rubber ingredients. 

Compared to coconut oil, the compound with sunflower oil is similar in the first minutes of the measurement, resulting in a similar cure rate and optimum cure time. However, the compound with sunflower oil reaches a much lower maximum torque value compared to coconut oil. Both plasticizers result in a much shorter scorch time and a faster cure rate compared to TDAE. The compound with cardanol shows a different curing behavior than the other samples, with a short scorch time of 6 min, and the lowest minimum and maximum torque values. Based on these observations, it can be stated that the bio-oils also influence the curing behavior. 

The low maximum torques of the samples with sunflower oil and cardanol are probable due to unsaturation in the tails of these plasticizers. This unsaturation in plasticizers can interfere with the curing reaction. Sulfur can react with the allylic position of the unsaturated bonds, creating crosslinks between the oil molecules, instead of between the polymer chains [18,19]. This leads to a lower CLD as can be observed in Table 4. The compounds with cardanol and sunflower oil show significantly lower CLD values compared to the compounds with fully saturated squalane and mainly saturated coconut oil. To verify the assumption that sulfur reacts with the unsaturation of the oil-consuming part of the vulcanization system, a model study was performed, which is discussed in Section 3.2 Model Study Sulfur and Plasticizers. Additionally, the TDAE compound shows similar CLD values as squalane and coconut oil. The exact structure of TDAE is unknown; it seems to be a mixture of noncarcinogenic aromatic and naphthenic substances. This result indicates that the number of reactive free double bonds in TDAE is indeed very low; aromatic rings will not react. 

The faster cure rate obtained for the sunflower oil and coconut oil could be due to the presence of ester groups within the plasticizer. These polar groups can interact with the silanol groups present on the silica surface and create hydrogen bonding [7]. The plasticizers can thereby shield the silica surface, resulting in fewer accelerators being adsorbed by this surface. This leads to more accelerators being available for the curing reaction, and hence the cure rate increases. In this way, both oils indirectly act as coactivators and speed up the vulcanization reaction in silica-filled compounds. Squalane does not contain functional groups and shows a more similar cure rate to TDAE. This indicates that the shielding of the silica surface does not occur for these plasticizers. 

The compound with cardanol results in a short scorch time due to the fact that it can function as an additional activator for the curing process. Alexander and Thachil [9] researched cardanol as a replacement for stearic acid. They found that cardanol can interact with zinc oxide, resulting in a Zn^2+^ salt. In contrast to their study, in this work, a relatively high concentration of cardanol is used in the compound (37.5 phr). Therefore, most of the zinc oxide may interact with either the stearic acid or the cardanol in the first minutes of the curing process, which results in the fast formation of zinc stearate or a “*zinc cardanol*” complex, enhancing the curing process and shortening the scorch time significantly. 

In general, the maximum torque is related to the CLD of the compound [20]. For sunflower oil and cardanol, the maximum torque values are much lower compared to TDAE, which can be explained by the low CLD values obtained for these compounds. The high maximum torque for coconut oil and the low maximum torque obtained for squalane can be partly explained by the differences in the CLD of these compounds compared to TDAE because the difference in CLD is only slight (Table 4). Therefore, it is likely that the difference in maximum torque between the compounds is influenced by other factors as well, for example, filler–filler interactions. This could indicate a better microdispersion for squalane and a worse dispersion for coconut oil compared to TDAE. Cured Payne effect measurements were performed to get insight into these filler–filler interactions. 

In addition, the minimum torque at the beginning of the measurement shows the influence of the different viscosities of the oils on the overall compound viscosity. Cardanol having the lowest molecular weight (302 g/mol) also results in the lowest minimum torque, while coconut (639 g/mol) and sunflower oil (881 g/mol) result in the highest minimum torques. 

#### 3.1.2. Cured Payne Effect

**Figure 2:**
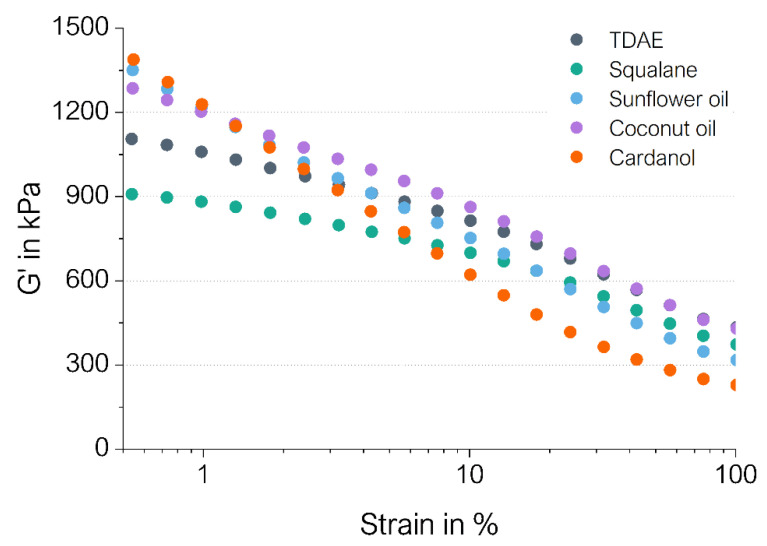
Cured Payne effect curves of the compounds.

Figure 2 shows the evolution of the storage modulus G′ with an increasing strain. Squalane results in the most similar shape of the G′ curve to TDAE, except that the G′ values of squalane are lower throughout the measurement. The plant-based plasticizers show a relatively linear decrease in G′ when increasing the strain, whereas the G′ values for TDAE and squalane follow a different trend. For these two plasticizers, the G′ decreases more rapidly at higher strain values than at low strains. The cured Payne effect (G′_0.56%_–G′_100%_) of the different compounds and the storage modulus at 100% strain (G′_100%_) are stated in Table 5.

The Payne effect is related to the amount of filler–filler interactions in a rubber compound. The filler network breaks when applying an increasing strain amplitude, which results in a decrease in the storage modulus (G′). The Payne effect is the difference between the G′ at low and high strain. A high value of G′_0.56%_–G′_100%_ is an indication of a high degree of filler–filler interactions in the compound [21]. Therefore, in highly filled systems, the Payne effect can also give insights into the microdispersion of the filler in the compound, where a high Payne effect is related to a worse microdispersion. 

The results in Table 5 show that sunflower oil and cardanol result in a much higher cured Payne effect compared to the other plasticizers. This indicates that the compounds with sunflower oil and cardanol exhibit a higher degree of filler–filler interactions. A possible explanation for this is based on the chemical nature of the plasticizers. Cardanol and sunflower oil contain unsaturated parts, which are likely to react with sulfur. In addition, the ester groups of sunflower oil can form hydrogen bonds with the silica. In this way, a more complex silica–oil–sulfur–oil–silica interaction can be formed, which leads to additional filler–filler interactions. Cardanol does not have ester groups, but it does contain hydroxyl groups. The polar hydroxyl group might interact with the silica as well, leading to similar additional filler–filler interactions to sunflower oil. This could explain the increase in cured Payne effect for these two plasticizers. Coconut oil may shield the silica particles as well due to the presence of ester groups, but this plasticizer cannot interact with other oil molecules due to its saturated nature. Therefore, in the compound with coconut oil, no additional filler–filler interactions are formed, leading to lower Payne effect values. 

Moreover, the potential interaction of the plant-based plasticizer with the silica surface could lead to a lower degree of silanization. If the plasticizer shields the silica surface, the interaction between silica and silane is hindered. A higher degree of silanization is related to a lower Payne effect [21]. Therefore, the high Payne effect could be explained by the worse silanization reaction. TDAE and squalane result in the lowest cured Payne effect values. Squalane is nonpolar, so it is not expected to interact with the polar silica surface like the plant-based plasticizers are assumed to do. Consequently, the squalane does not interfere with the silanization reaction between the TESPD and the silica. The low Payne effect obtained for squalane might be caused by an improved silanization reaction. A similar explanation may hold for TDAE as well. However, TDAE possibly still interacts to some extent with the polar silica, leading to the slightly higher Payne effect of TDAE compared to squalane. Potentially, the difference in maximum torque as shown in Figure 1 is also influenced by the few additional filler–filler interactions observed for the reference compound with TDAE compared to squalane. 

The G′_100%_ is directly related to the in-rubber structure, hydrodynamic effects and the polymer network [22]. These last two contributions are stable over the entire strain range, while the in-rubber structure is influenced by the changing filler–filler and filler–polymer interactions when increasing the strain. In terms of the G′_100%_, it can be observed that coconut oil is similar to TDAE, squalane displays a slightly lower value and, for sunflower oil, an even lower value is obtained. Cardanol results in the lowest G′_100%_ value. Comparing these values to the CLD values in Table 4, a similar trend in CLD as in G′_100%_ can be observed. Likely, the differences in G′_100%_ are due to differences in the polymer network. Moreover, as explained previously, the bio-based oils possibly result in a lower degree of silanization, and so fewer filler–rubber interactions occur. This would lead to lower in-rubber structures and could explain the lowest G′_100%_ values for cardanol and sunflower oil. 

#### 3.1.3. Stress–Strain Behavior 

**Figure 3:**
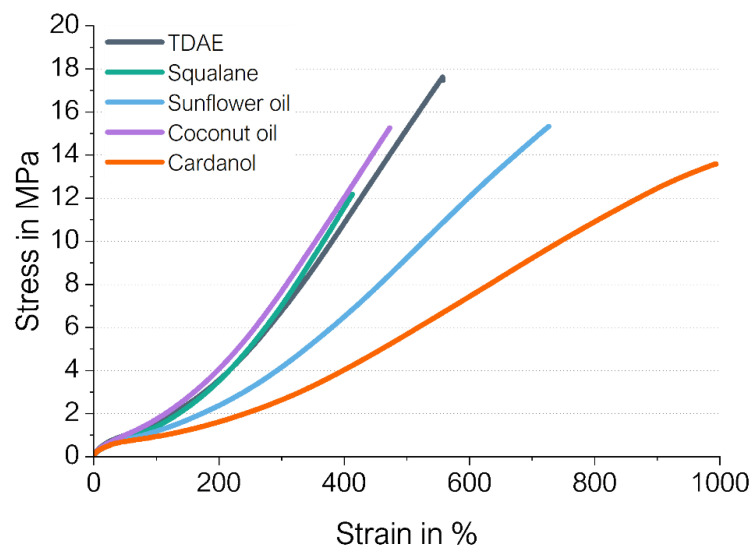
Stress–strain behavior of the compounds.

Figure 3 shows the stress–strain curves, and Table 6 shows the tensile strength, elongation at break and reinforcement index (modulus at 300% strain divided by the modulus at 100% strain) of the studied compounds. 

For all bio-based alternatives, the tensile strength decreases. Squalane shows a similar curve to TDAE, except for a much lower elongation at break and therefore tensile strength. 

For cardanol and sunflower oil, a severe increase in elongation at break is obtained as well as a lower tensile strength and lower reinforcement index. These values can be again explained by the lower crosslinking density of these compounds compared to TDAE caused by the possible reaction of the vulcanization system with the unsaturated double bonds of both oils. Therefore, a reduce polymer network or crosslinking density, respectively, can be expected. The model study of Section 3.2 further elaborates on this theory. Furthermore, the fewer filler–rubber interactions, as discussed in the previous subsection, contribute as well to an overall lower reinforcement effect, which is indeed observed for these two bio-oils.

Coconut oil has the same reinforcement index as TDAE, but the tensile strength and elongation at the break of coconut oil are both lower. It might be that the crosslinks formed in the compound with coconut oil are slightly shorter compared to those formed in the reference compound with TDAE. Because the bio-based plasticizers may be able to shield the silica surface, more accelerators would be available to enhance the curing reaction. Therefore, there will be a shift in the ratio between the accelerator (A) and sulfur (S) (the A/S ratio). An increase in the A/S ratio by keeping the sulfur content constant might result in a slightly more efficient curing process increasing the total amount of crosslinks formed. Additionally, compounds with an efficient curing system result in more monosulfidic bonds, which means shorter crosslinks [23]. The slightly higher crosslink density based on the shorter crosslinks in coconut oil compared to TDAE could explain the slight decrease in tensile strength and elongation at break. 

Squalane results in a similar reinforcement index to coconut oil and TDAE but a much lower tensile strength and elongation at break. Squalane has a mainly linear nonpolar chemical structure with a relatively low molecular weight (Table 1), which means that the substance cannot enhance the strength of the compound by forming additional interactions between the plasticizer and the other rubber ingredients.

#### 3.1.4. Dynamic Mechanical Analysis

**Figure 4:**
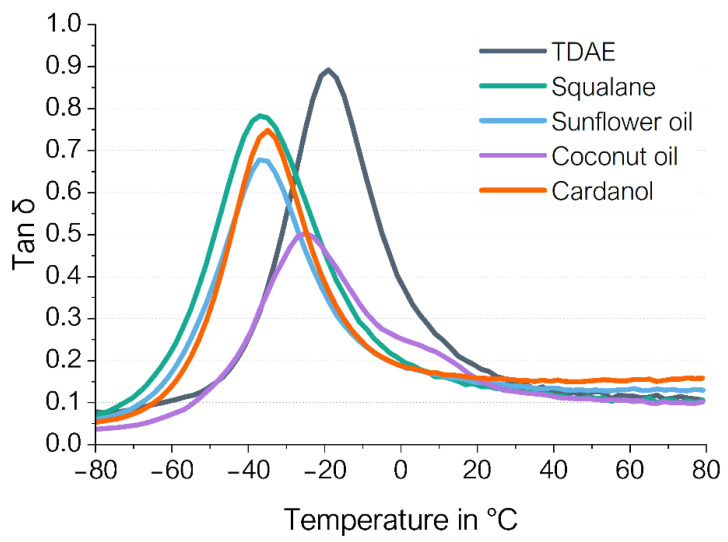
Tan δ curves of the compounds.

Figure 4 shows the tan δ curves obtained from the dynamic mechanical analysis (DMA). These results can be used to obtain indicators for tire properties. For example, the tan δ at 60 °C is an indication of the rolling resistance, whereas the tan δ at 0 °C indicates the wet grip performance of the tire [24]. In the DMA test, a low rolling resistance indicator and a high wet grip indicator are preferred. The tan δ peak gives insight into the glass transition temperature (Tg) of the compound. Table 7 displays the values of the glass transition temperature (Tg), the tan δ peak height, the tan δ at 0 °C and the tan δ at 60 °C. 

As can be seen in Figure 4, all bio-based alternatives and squalane shift the tan δ peak to lower temperatures, which means that these compounds have a lower Tg than TDAE. Because of the shift in Tg to low temperatures, the wet grip indicator of all plasticizer alternatives is also much lower than that for TDAE. The rolling resistance indicator is slightly lower for coconut oil and squalane compared to the reference with TDAE, whereas sunflower oil and cardanol result in increased rolling resistance indicators. 

In the case of plasticizers, lower Tg values indicate less internal friction, which might be caused by an increased free volume in the material. Squalane, cardanol and sunflower oil have a lower softening point compared to coconut oil and TDAE (Table 1). Therefore, these alternatives are less viscous at low temperatures. It could be that these plasticizers are more mobile and easier to migrate to the free volume at low temperatures. Therefore, the Tg of the cardanol and sunflower oil is lower, compared to the coconut oil and TDAE. The shift of the tan δ peak of coconut oil is less severe than the other plasticizer alternatives. In addition, a shoulder can be observed for coconut oil between 0 and 20 °C. This can be explained by the poorer compatibility of the coconut oil with the used polymers compared to the other plasticizers. When materials are fully miscible, the blend will have one single Tg. In the case of immiscible blends, the Tg values of the separate components will not change, and two single peaks will be observed corresponding to each ingredient [25]. Table 1 shows that coconut oil has a lower total Hansen solubility parameter compared to TDAE, cardanol and sunflower oil. Therefore, coconut oil is potentially less compatible with the used polymers compared to the other plasticizers. Additionally, coconut oil has a higher softening temperature [26]. The second peak between 0 and 20 °C could be an indication of the softening behavior. 

The wet grip indicator shows severe differences between the plasticizers. All bio-based alternatives and squalane show a much lower value compared to the reference with TDAE. Sunflower oil and cardanol especially result in a low tan δ at 0 °C with a value of 0.191 (Table 7). The low values can be explained by the shift in Tg obtained for all bio-based replacements. Because of this shift, the highest point of the tan δ peak lies at lower temperatures. Therefore, the tan δ decreases to lower values at 0 °C compared to TDAE.

The rolling resistance indicator, which is the tan δ at 60 °C, is similar for the coconut oil and squalane but increased for the sunflower oil and cardanol. Values of 0.152 and 0.131 are obtained for the cardanol and sunflower oil, respectively (Table 7). More energy is dissipated for these samples, due to more filler–filler interactions. This is in line with the higher cured Payne effects obtained for cardanol and sunflower oil compared to TDAE, squalane and coconut oil. This indicates that the internal friction of the samples with cardanol and sunflower oil is higher, which results in a higher rolling resistance. Additionally, the release of occluded rubber when the weak filler–filler network breaks due to dynamical deformation might contribute to the higher rolling resistance indicator as well. 

### 3.2. Model Study Sulfur and Plasticizers

**Figure 5:**
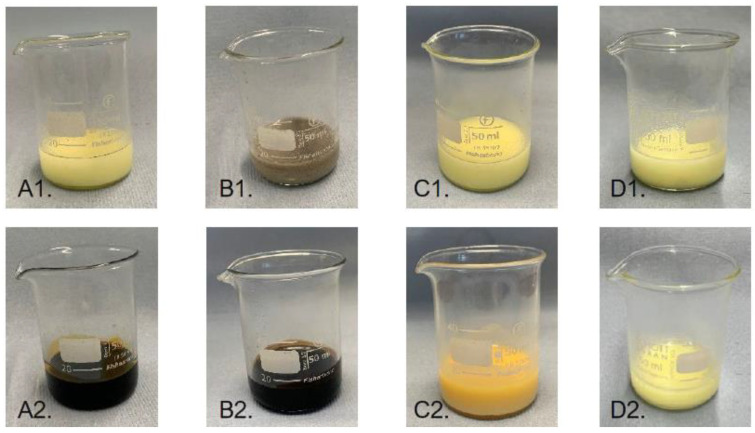
Sulfur mixed at RT with sunflower oil (**A1**), cardanol (**B1**), coconut oil (**C1**) and squalane (**D1**). Sulfur in sunflower oil (**A2**), cardanol (**B2**), coconut oil (**C2**) and squalane (**D2**) after mixing for 1 h at 160 °C.

To verify the assumption that sulfur reacts with the unsaturation of the oil, a model study was performed by just mixing sulfur and oil. This study was performed as a proof of concept to support the findings obtained in the cure curves and the CLD values (Table 4). An excess of sulfur was mixed with the plant-based alternatives for 1 h at the curing temperature of 160 °C. Figure 5 shows the mixtures before and after the reaction.

Figure 5(A2,B2) corresponds to the mixtures with sunflower oil and cardanol and shows dark colors. Moreover, it was evaluated that the viscosity of these mixtures was higher compared to the mixtures before heating (Figure 5(A1,B1)). These differences indicate that a reaction between the sulfur and the oil took place. In Figure 5(C2), it can be seen that the mixture of sulfur and coconut oil remained light and yellow after mixing at elevated temperatures. After cooling the mixture down, clear sulfur particles were still visible, while this was not observed for the mixtures with cardanol and sunflower oil. Thereby, the viscosity of the mixture with coconut oil stayed the same. Taking the chemical nature of the plasticizers into consideration, it can be stated that the color and viscosity change is not due to the ester groups because these are present in both sunflower and coconut oil. The only difference between these two plasticizers is the unsaturated bonds in the tail structures. Therefore, it can be concluded that sulfur can indeed react with unsaturated plasticizers. Figure 5(D1,D2) shows the mixtures with sulfur and squalane at room temperature and after heating, respectively. Both mixtures have the same color. Furthermore, similar to coconut oil, for squalane, the sulfur particles were still visible after the reaction at elevated temperatures. Because squalane does not have double bonds or other chemical functional groups, no reactions were expected. The mixture with squalane and sulfur is an additional proof that the sulfur does not react with the fully saturated plasticizers. 

The unsaturated bonds within cardanol and sunflower oil bear the possibility to oxidize or degrade after being exposed to elevated temperatures during mixing and vulcanization. Therefore, supplementary to the previous model study, the pure oils themselves without the addition of sulfur were heated at 160 °C for 1 h as well. It was expected that if degradation and oxidation take place, the color or viscosity of these pure oils will change. However, no changes in any saturated and unsaturated oils could be observed excluding the altering of their chemical structure. This finding confirms that the changes observed in the mixtures with sulfur are due to chemical reactions with this curing agent. This model study confirms the abovementioned assumption that the differences obtained in curing behavior (Figure 1) for the saturated and unsaturated oils are due to reactions between the unsaturation of the oils and the curing agent. 

## 4. Conclusions

Three plant-based plasticizers and squalane were used as TDAE replacements in a simplified tire-tread formulation. The cure curves, cured Payne effect, stress–strain behavior and tan δ curves of all the studied compounds were analyzed. The replacement of TDAE with bio-based oils showed that using oils in a rubber compound does influence the material properties. For instance, it was observed that the plant-based alternatives decrease the scorch time compared to TDAE. The alternatives containing unsaturated chains (cardanol and sunflower oil) show much lower maximum torque values in the cure curves, lower crosslink densities measured with equilibrium swelling experiments and higher cured Payne effect results. With a model study, the interaction between oils and sulfur was further investigated, and it was verified that the unsaturated oils do interact with sulfur, while this is not the case for the fully saturated oils. The tan δ curves show severe shifts of the glass transition temperature to a lower temperature for sunflower oil and cardanol compared to TDAE and coconut oil. Most likely, this shift is influenced by the differences in glass transition temperature and compatibility of the plasticizer with the used polymers. In addition to this, the wet grip indicator was clearly influenced negatively by the bio-based oils. However, it is questionable if the tan δ at 0 °C is a proper indicator of the wet grip by changing the ingredients [27]. Further studies, for example with the LAT100, should be performed to verify these values. Although all three oils show some potential, they are not suitable as a one-to-one replacement for TDAE in a tire-tread compound. Research needs to be performed to find bio-based alternatives that result in similar tan δ curves to TDAE and that result in similar reinforcement behavior. One approach could be to adjust the vulcanization system for the unsaturated oils leading to a possible increase in crosslinking density and therefore performance. Furthermore, it could be attempted to oxidize the unsaturated bonds prior to the mixing to decrease their interaction with the vulcanization system. Finally, oil-blend systems or other ingredients like resins, for example, could be added to partially or fully replace the currently used fossil-based TDAE oil and hence increase the sustainability of the rubber compound.

Squalane was used in this study to investigate the influence of TDAE on the compound in more detail. Squalane has a fully saturated aliphatic structure; therefore, it was assumed that the interactions with and influences on the other rubber ingredients were limited for this substance. Clear differences between squalane and TDAE were observed, as TDAE results in a higher maximum torque, a higher cured Payne effect, a higher tensile strength and a higher glass transition temperature. This shows that TDAE does not only act as a plasticizer. It interacts additionally with other rubber ingredients and contributes in this form to the reinforcement of the compound. 

## Figures and Tables

**Table 1:** Characteristics and chemical structures of used plasticizers.

Plasticizer	Mw in g/mol	ρ at 25 °C in g/cm^3^	MP in °C	HSP in MPa^1/2^	Chemical Structure	
TDAE	X	0.95	27 *	δ_tot_ = 18.9	X	
Squalane	423	0.81	−38	δ_D_ = 15.9 δ_P_ = 0.1 δ_H_ = 0.1 δ_tot_ = 15.9	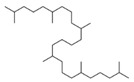	
Sunflower oil	~881	0.92	−17	δ_D_ = 16.5 δ_P_ = 1.7 δ_H_ = 3.6 δ_tot_ = 17.0	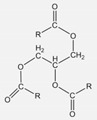	where R = C16:0 (5%), C18:0 (6%), C18:1 (30%), C18:2 (59%)
Coconut oil	~639	0.90	23–27	δ_D_ = 16.3 δ_P_ = 2.3 δ_H_ = 2.6 δ_tot_ = 16.7	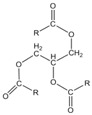	where R = C8:0 (8%), C10:0 (7%), C12:0 (49%), C14:0 (8%), C16:0 (8%), C18:0 (2%), C18:1 (6%), C18:2 (2%)
Cardanol	~302	0.93	−30	δ_D_ = 17.4 δ_P_ = 2.2 δ_H_ = 5.8 δ_tot_ = 18.5	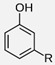	where R = C_15_H_27_

Data of TDAE were obtained from [14]. Other data were calculated with the Hansen solubility parameter in Practice (HSPiP) software (5th edition, version 5.4.04) or were provided by the supplier. * TDAE does not have a “melting point”, but a pour point (the temperature at which an oil starts to be able to flow). In addition, the HSP values for the SSBR/BR blend were calculated (δ_tot_ = 18.9 MPa^1/2^, δ_D_ = 18.5 MPa^1/2^, δ_P_ = 0.9 MPa^1/2^, δ_H_ = 1.7 MPa^1/2^).

**Table 2:** Rubber formulations.

Ingredient	TDAE	Squalane	Sunflower Oil	Coconut Oil	Cardanol
S-SBR	80	80	80	80	80
BR	20	20	20	20	20
Silica	80	80	80	80	80
TESPD	6.2	6.2	6.2	6.2	6.2
TDAE	37.5	X	X	X	X
Squalane	X	37.5	X	X	X
Sunflower oil	X	X	37.5	X	X
Coconut oil	X	X	X	37.5	X
Cardanol	X	X	X	X	37.5
Stearic acid	2.5	2.5	2.5	2.5	2.5
Zinc oxide	2.5	2.5	2.5	2.5	2.5
Sulfur	1.4	1.4	1.4	1.4	1.4
TBBS	2	2	2	2	2
DPG	1.5	1.5	1.5	1.5	1.5

All values are in phr.

**Table 3:** Mixing procedure.

Time	Action
min:s	Stage 1: preheating 80 °C, 70 rpm, fill factor 72%
0:00	Addition of rubber
1:00	Addition of 2/3 silica, 2/3 silane
2:30	Addition of 1/3 silica, 1/3 silane, ZnO, stearic acid and plasticizer
4:00	15 s sweep
4:15	Increase in torque (increase temperature to 130 °C)
7:00	Stop mixing (reaching 140 °C)
min:s	Stage 2: preheating 80 °C, 80 rpm, fill factor 69%
0:00	Addition of elastomer masterbatch
0:50	Addition of DPG
1:00	Increase in torque (increase temperature to 130 °C)
5:00	Stop mixing (reaching 140 °C)
min:s	Stage 3: preheating 50 °C, 50 rpm, fill factor 66%
0:00	Addition of elastomer masterbatch, curatives (sulfur and TBBS)
3:00	Stop mixing

**Table 4:** Additional data about the curing behavior.

Compound	t90 in min	t2 in min	MH in dNm	ML in dNm	CLD in mol/cm^3^
TDAE	37	14	12.6	1.3	184 ± 2
Squalane	37	14	10.8	1.1	174 ± 5
Sunflower oil	33	11	11.6	1.2	129 ± 3
Coconut oil	31	11	13.8	1.4	194 ± 3
Cardanol	36	6	10.3	0.8	92 ± 1

**Table 5:** Cured Payne effect data.

Compound	G′_0.56%_–G′_100%_ in kPa	G′_100%_ in kPa
TDAE	671	434
Squalane	536	373
Sunflower oil	1034	317
Coconut oil	857	429
Cardanol	1160	228

**Table 6:** Additional data on the stress–strain behavior.

Compound	Tensile Strength in MPa	Elongation at Break in %	Reinforcement Index M300/M100
TDAE	16.7 ± 1.7	510 ± 40	4.8 ± 0.3
Squalane	12.1 ± 1.7	420 ± 40	4.8 ± 0.3
Sunflower oil	15.6 ± 0.3	720 ± 7	3.5 ± 0.1
Coconut oil	14.4 ± 1.0	460 ± 20	4.4 ± 0.1
Cardanol	13.3 ± 0.6	990 ± 30	2.7 ± 0.1

**Table 7:** Data obtained during DMA measurements.

Compound	Tg in °C	Tan δ Peak Height	Tan δ at 0 °C	Tan δ at 60 °C
TDAE	−19	0.892	0.403	0.116
Squalane	−39	0.783	0.208	0.106
Sunflower oil	−37	0.678	0.191	0.131
Coconut oil	−25	0.503	0.256	0.104
Cardanol	−35	0.749	0.191	0.152

## Data Availability

The raw data supporting the conclusions of this article will be made available by the authors on request.
